# Low profile multi-layered invisibility carpet cloak using quantum dot core–shell nanoparticles

**DOI:** 10.1038/s41598-023-30389-2

**Published:** 2023-03-01

**Authors:** Amin Monemian Esfahani, Leila Yousefi

**Affiliations:** School of Electrical and Computer Engineering, College of Engineering, University of Tehran, Tehran, 1417614411 Iran

**Keywords:** Electrical and electronic engineering, Metamaterials, Nanoparticles

## Abstract

In this paper, a method to reduce the profile of layered carpet cloaks is proposed. We analytically prove and numerically demonstrate that using a Low Index Material (LIM), a material with a relative dielectric constant smaller than 1, in construction of carpet cloaks can remarkably reduce their profiles. Using the proposed technique, a carpet cloak consisting of alternating LIM and silicon layers is designed to provide invisibility at visible wavelengths. The designed cloak has a profile that is 2.3 times smaller than a carpet cloak without LIM layers. To realize low index materials at optical wavelengths, silver-coated CdSe/CdS quantum dots dispersed in a polymer host are used. Quantum dots are utilized to compensate the loss of Silver and to achieve a low index medium with neglectable loss. The designed low profile carpet cloak is numerically analyzed showing a good performance for a wide range of incident angles which is the advantage of the proposed structure in comparison with metasurface-based carpet cloaks which work only for a very narrow range of incident angles.

## Introduction

Invisibility cloaks now have been turned into a scientific reality thanks to the pioneering work done by Pendry and Leonhardt^1^. That first pioneering work inspired many researchers around the world to develop invisibility cloaks operating at different frequency regimes, using transformation optics theory^2–6^. Transformation optics provides a precise design tool for manipulation of electromagnetic (EM) waves, which can be used for different applications including designing invisibility cloaks^2–10^. The first invisibility cloaks designed based on transformation optics were difficult, or even impossible in some cases, to be realized, due to requiring materials with strange specifications such as singularity and/or high level of dispersion in Electromagnetic properties^1–7^. To address this problem, several reduced invisibility cloaks with simplified constitutive parameters have been proposed and realized so far^2,11–18^. One of the most successful schemes proposed so far, is a special cloaking technique, called the “carpet cloak”^13–18^.

Carpet cloaks are designed based on quasi-conformal coordinate transformation^13–18^ and can conceal an object under a curved reflecting surface (called as carpet) from an observer locating above the object. In this technique, the invisibility cloak imitates the reflection of a flat surface meaning that they are designed in such a way that reflect the incident waves exactly as they are reflected from a flat surface^13–18^. Therefore, the observer receiving the reflected waves assumes that there is only a flat surface (ground) there, and cannot detect objects on the ground. Carpet cloaks have been successfully realized in both microwave and optical frequency ranges^16–18^. In order to realize the homogeneous anisotropic material required for developing carpet cloaks, one of the best methods proposed so far, is to use alternative layers of isotropic dielectrics^12,19–22^. However, this method suffers from large size of the cloaks, meaning that in order to conceal an object, we need a carpet cloak with a height much bigger than the object itself, which makes this method non-practical for most of real-world applications. To solve this problem, metasurface-based cloaks were proposed which are extremely thin^23–25^. Metasurfaces which are 2D version of metamaterials^26–32^ have recently found many applications including realization of photonic topological insulators^26^, light trapping in thin film solar cells^28,29^, optical beam steering^30^, and sub-wavelength imaging^31,32^. Using metasurfaces for developing invisibility cloaks, can remarkably reduce the thickness of the resultant cloaks, however, these types of cloaks have a very high sensitivity to the incident angle, and operates well only for angles very close to normal incidence^23–25^.

Zero refractive index medium has found some applications in reducing the thickness of the carpet cloaks^33^. The height ratio of the carpet cloak to the object in the proposed structure^33^ is about 6.7, meaning that the thickness of the carpet cloak has not been reduced remarkably.

Here, to address this issue, we propose a method to decrease the height of the carpet cloaks. In the proposed method, we use Low Index Material (LIM) layers with a relative dielectric constants smaller than 1, to develop our carpet cloak. We first analytically prove that using a low index material in design of carpet cloaks can significantly reduce the height of the cloak and then numerically validate our claim. The designed structure consists of alternating low index layers and silicon (Si) layers providing invisibility at the wavelength of $534\,\,\mathrm{ nm}$. To provide a low index material at visible wavelengths with a negligible loss, we have used silver-coated CdSe/CdS quantum dots randomly distributed inside a polymer host. To calculate the effective permittivity of the quantum dots, we use Clausius–Mossotti relations, and then numerically validate the analytical results using a full wave characterization method. Both analytical and numerical results show that the designed nano-particles provide a low index material with almost no loss. Here, quantum dots act as a gain material compensating the loss provided by the Silver, and thus resulting in a low index layer with almost no loss. Using alternating Silicon layers and layers containing quantum dots, we design our carpet cloak and numerically analyze it. In numerical analysis, full wave numerical simulation is used to find the response of the structure to the incident light with different incident angles.

Our numerical results show that the proposed carpet cloak can conceal the object for all incident angles in the range of ${0}^{^\circ }$–${60}^{^\circ }$. This is the main advantage of the proposed cloak over other thin carpet cloaks such as metasurface-based ones^23–25^, which typically operate for incident angles in the range of ${0}^{^\circ }$–${15}^{^\circ }$. Comparing to conventional multi-layered carpet cloaks, they suffer from large profiles which makes the realization of the structures so complicated^12,19–22^. The height ratio of the carpet cloak to the object in these designs^12,19–22^ is more than 5, while this ratio is 2 in our proposed carpet cloak. Meaning that the thickness of the cloak has been remarkably reduced. Furthermore, the background medium used in these structures is not Air which makes them impractical. However, by designing the specific parameters and using low index layers in the proposed structure, the background medium of our structure is Air making it realizable and practical.

In this paper, first, we theoretically prove how using materials with a relative dielectric constant smaller than one can significantly reduce the carpet cloak height. Then, the realization of a low index slab using quantum dots is explained and the resultant electromagnetic properties are calculated analytically and validated numerically. Then, the proposed cloak is numerically analyzed and its responses to the light beams with different incident angles, are presented and discussed. Finally, we summarize and conclude the paper.

## Proposed structure and theoretical background

**Figure 1:**
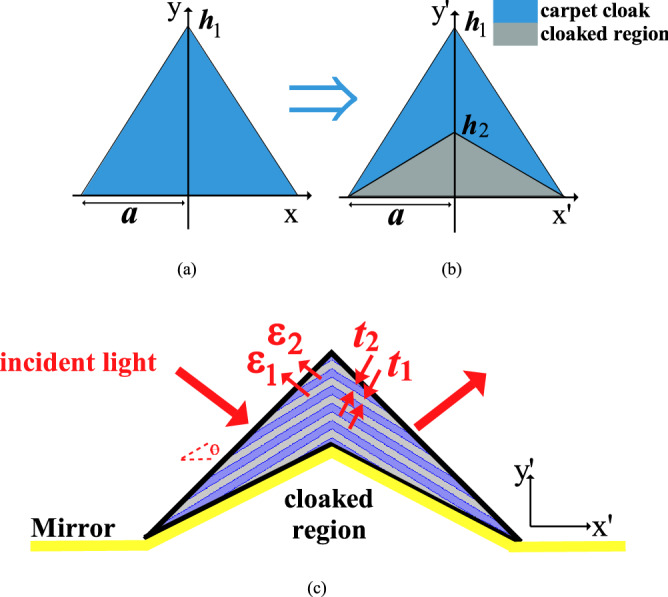
Scheme of the coordinate transformation (**a**) virtual space, (**b**) physical space. The cloaked region is shown in gray. (**c**) The schematic of a carpet cloak realized by alternating dielectric *1* and *2*. Any object in the cloaked region is invisible to incident light.

To design the carpet cloak, a linear optical transformation^7,34,35^ is used, as schematically illustrated in Fig. 1. As shown in this figure, in this transformation, a virtual space $(x,y,z)$ with a triangular cross-section in the x–y plane is squeezed into a blue region in the physical space $({x}^{{\prime}},{y}^{\mathrm{^{\prime}}},{z}^{{\prime}})$ with quadrilateral cross-section, where the lower gray triangle is supposed as the cloaked region^7^. Under this transformation, the light shining on the structure, will be squeezed into the upper quadrilateral area, which is called the carpet cloak, keeping the lower triangle unaffected by the light and therefore invisible from the incoming light. Since Maxwell’s equations are coordinate invariant, this type of coordinate transformation could be realized by engineering the properties of the materials exist in the physical space^7^. This coordinate transformation can be achieved by defining ${({x}^{{\prime}},y}^{{\prime}})$, the coordinates of the physical space as:

1 $${{y}^{\prime}}={\frac{{h}_{1}-{h}_{2}}{{h}_{1}}}y+{\frac{{h}_{2}}{a}}\left(a-{\left|x\right|}\right),\quad {{x}^{\prime}}=x$$

where ($$x,y)$$, represent the coordinates of the virtual space, $${h}_{1}$$ and $${h}_{2}$$ are the height of the carpet cloak and the object, respectively, and $$a$$ is half of the lateral dimension of the cloaked region (see Fig. 1c). Now, By using the procedure of transformation optics, the material parameters can be derived^7^. For a light with TM polarization, with the magnetic field perpendicular to the $$x$$–$$y$$ plane, the transformation shown in (1) results to the following nonmagnetic ($$\mu{^{\prime}}={\mu }_{0}$$) material for the carpet cloak^7^:

2 $${\overline{\overline{\varepsilon }}}^{{\prime}}={\varepsilon }_{0}\left(\begin{array}{ccc}{\left[{h}_{1}/\left({h}_{1}-{h}_{2}\right)\right]}^{2}& \frac{{h}_{2}}{a}{\left[{h}_{1}/\left({h}_{1}-{h}_{2}\right)\right]}^{2}& 0\\ \frac{{h}_{2}}{a}{\left[{h}_{1}/\left({h}_{1}-{h}_{2}\right)\right]}^{2}& {\left(\frac{{h}_{2}}{a}\right)}^{2}{\left[{h}_{1}/\left({h}_{1}-{h}_{2}\right)\right]}^{2}+1& 0\\ 0& 0& {\varepsilon }_{z}\end{array}\right)$$

It should be noted that, if the assumption of TM polarization is not considered, both permittivity and permeability should be engineered^7^. Designing materials with desired magnetic properties at optical frequencies, is still a challenge and many researchers around the world are working on it. Therefore, at this stage, the proposed cloak is designed to operate only for a TM polarized light. Equation (2) defines an anisotropic dielectric which is spatially invariant. The dielectric optical axis is rotated by a certain angle $\theta$ to the $z$-axis. Through coordinate rotation, a diagonal permittivity tensor ${\overline{\overline{\varepsilon }}}^{c}$ which is related to the required permittivity tensor in Eq. (2) can describe this anisotropic dielectric in the local coordinate system as^6^:

3 $${\overline{\overline{\varepsilon }}}^{{{\prime}}}=\left(\begin{array}{ccc}\mathrm{cos}\theta & -\mathrm{sin}\theta & 0\\ \mathrm{sin}\theta & \mathrm{cos}\theta & 0\\ 0& 0& 1\end{array}\right)\left(\begin{array}{ccc}{\varepsilon }_{x}^{c}& 0& 0\\ 0& {\varepsilon }_{y}^{c}& 0\\ 0& 0& {\varepsilon }_{z}^{c}\end{array}\right)\left(\begin{array}{ccc}\mathrm{cos}\theta & \mathrm{sin}\theta & 0\\ -\mathrm{sin}\theta & \mathrm{cos}\theta & 0\\ 0& 0& 1\end{array}\right)$$

By using diagonalization of the parameters, the principal values of the permittivity tensor ${\varepsilon }_{x}^{c}, {\varepsilon }_{y}^{c}$ and ${\varepsilon }_{z}^{c}$ can be calculated. If the incident wave is TM polarized, ${\varepsilon }_{z}^{c}$ can be assumed arbitrary as ${\varepsilon }_{z}^{c}={\varepsilon }_{x}^{c}$. Therefore, a birefringent dielectric crystal defined by ${\varepsilon }_{x}^{c}$ and ${\varepsilon }_{y}^{c}$ can realize the desired carpet cloak. One of the best methods can be used to realize the birefringent dielectric is through multilayer of alternating dielectric 1 and dielectric 2 (as shown in Fig. 1c). According to Effective Medium Theory^36,37^, the dielectric constants, ${\varepsilon }_{y}^{c}, {\varepsilon }_{x}^{c}$ required for the carpet cloak, can be achieved using a layered structure as^6,7^:

4 $${\varepsilon }_{z}^{c}={\varepsilon }_{x}^{c}=\frac{{\varepsilon }_{1}+\eta {\varepsilon }_{2}}{1+\eta } , {\varepsilon }_{y}^{c}=\frac{(1+\eta ){\varepsilon }_{1}{\varepsilon }_{2}}{\eta {\varepsilon }_{1}+{\varepsilon }_{2}}$$

where ${\varepsilon }_{1}$ and ${\varepsilon }_{2}$ are the permittivity of dielectric 1, and dielectric 2, respectively and $\eta =\frac{{t}_{2}}{{t}_{1}}$ is the ratio of thicknesses of two isotropic dielectrics (see Fig. 1c).

Here, in this paper, our goal is to decrease the height of the carpet cloak and make it as thin as possible. In the following parts, we analytically prove that this goal can be achieved by using a low index material for one of the layers used to construct the carpet cloak. To prove this, we use the equality of eigenvalues of tensor ${\overline{\overline{\varepsilon }}}^{{^{\prime}}}$ with permittivity values; ${{\lambda }_{1}=\varepsilon }_{x}^{c}$ and ${{\lambda }_{2}=\varepsilon }_{y}^{c}$, where ${\lambda }_{1}$ and ${\lambda }_{2}$ are derived from equation of (det (${\overline{\overline{\varepsilon }}}^{\mathrm{^{\prime}}}-\lambda I)=0$)). Dividing ${\lambda }_{1}$ by ${\lambda }_{2}$, we will have:

5 $$\frac{{\lambda }_{1}}{{\lambda }_{2}}=\frac{H+{P}^{2}H+1+\sqrt{{(H+{P}^{2}H+1)}^{2}-4H}}{H+{P}^{2}H+1-\sqrt{{(H+{P}^{2}H+1)}^{2}-4H}}$$

where $$H={\left(\frac{{h}_{1}}{{h}_{1}-{h}_{2}}\right)}^{2}$$ and $$P=\frac{{h}_{2}}{a}.$$

To have a thin carpet cloak, it is required that ${h}_{1}\to {h}_{2},$ resulting $H\to \infty$. That will result in $\frac{{\lambda }_{1}}{{\lambda }_{2}}$ tends to infinity. On the other hand, we have:

6 $$\frac{{\lambda }_{1}}{{\lambda }_{2}}=\frac{{\varepsilon }_{x}^{c}}{{\varepsilon }_{y}^{c}}=\frac{(1+\eta \frac{{\varepsilon }_{2}}{{\varepsilon }_{1}})(1+\eta \frac{{\varepsilon }_{1}}{{\varepsilon }_{2}})}{{(1+\eta )}^{2}}$$

According to (6) for the ratio of $\frac{{\lambda }_{1}}{{\lambda }_{2}}$ to tend to infinity, we require that eighter $\frac{{\varepsilon }_{2}}{{\varepsilon }_{1}}\to \infty$ or $\frac{{\varepsilon }_{1}}{{\varepsilon }_{2}}\to \infty$. Therefore, in order to have a thin layered carpet cloak, dielectric layers with a large permittivity difference should be used in construction of the cloak. In this paper, we propose to use a material with the relative dielectric constant smaller than 1 for one of the layers, to make this ratio as big as possible, resulting in the shrink of height of the resultant carpet cloak.

**Figure 2:**
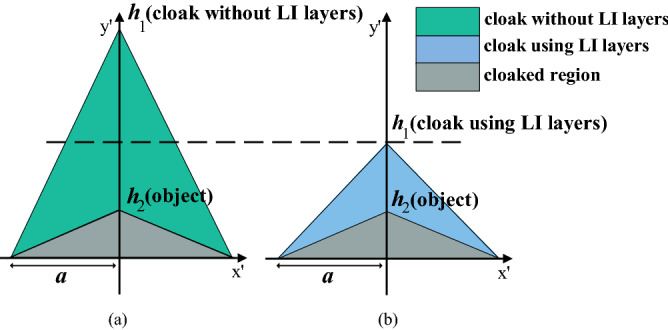
Comparison of two designed carpet cloaks. (**a**) The cloak without low index layers and (**b**) the cloak using low index layers. The cloak thickness is remarkably reduced in (**b**).

As a proof of concept, we design two carpet cloaks (one with a LIM and the other without a LIM) to hide the same object and compare them with each other. The object in both designs is assumed to have no variation in the z direction with a cross section to be a triangle with ${h}_{2}/a=0.5$ (see Fig. 2), and the both cloaks are designed to operate at the wavelength of $\lambda =534 \,\,{\rm nm}$. In the first carpet cloak, Silicon (${\varepsilon }_{r1}=17.1$) and Air are used as the alternating layers, while in the second carpet cloak, Silicon and a LIM (${\varepsilon }_{r2}=0.55$) are used to construct the carpet cloak.

Using Eqs. (2–4), the other parameters of the cloaks which are the height of the cloak ${h}_{1}$, the ratio of thickness of alternating layers, $\frac{{t}_{2}}{{t}_{1}}$, and the tilt angle, $\theta$, are calculated for both designs and shown in Table 1.

**Table 1:** Design parameters of two cloaks, with and without low index layers, are compared with each other in this table.

	Cloak without a LIM	Cloak using LIM
Background permittivity (${\varepsilon }_{b}$)	1.8	1
$\eta =\frac{{t}_{2}}{{t}_{1}}$	4.1	2.55
$\theta$	41.07	31.7
${\varepsilon }_{r1}$	17.17 (Si)	17.17 (Si)
${\varepsilon }_{r2}$	1 (air)	0.55 (LIM)
${t}_{1}$	15 nm	15 nm
${h}_{1}/a$	2.3	1

The two cloaks are shown and compared in Fig. 2. As shown in this figure, the height of the structure which does not use low index layers is 2.3 times bigger than the one that uses these layers, therefore, using low index layers can significantly reduce the thickness of the carpet cloaks designed based on layered structures.

Also, as indicated in this table, the designed background permittivity of the cloak using low index layers is 1 (air) which simplifies the realization of the structure in comparison with the cloak without low index layers which requires a background material with the relative permittivity of 1.8. This problem in realization of layered carpet cloaks has been also reported in previous works^6,7^.

## Realization of low index materials using quantum dots

In this paper, to realize the low index layer, we propose to use core–shell nanoparticles dispersed in Polyethylene glycol (PEG) as host medium with relative permittivity of (${\varepsilon }_{host}=2.11$). For the shell, we have used Silver, which provides negative permittivity at the visible wavelengths, and its combination with the core and host medium, can provide a low index material. The core has been selected as CdSe/CdS quantum dots which are proven to act as a gain medium^38,39^ when exited by an UV diode laser ($\lambda =405\,\,\mathrm{ nm}$) with a numerical aperture of NA = 1.40^40^. We have used these specific quantum dots to compensate the loss provided by Silver. If quantum dots are not used, the light going through the layers of the carpet cloak will experience a non-neglectable loss that significantly decreases its intensity and therefore, the carpet cloak won’t act efficiently. The CdSe/CdS quantum dots used as the core are fabricated and characterized in^39^. For the shell, Silver was chosen because most of the core–shell nanoparticles fabricated in previously reported works, has used Silver as shell^38,41,42^.

**Figure 3:**
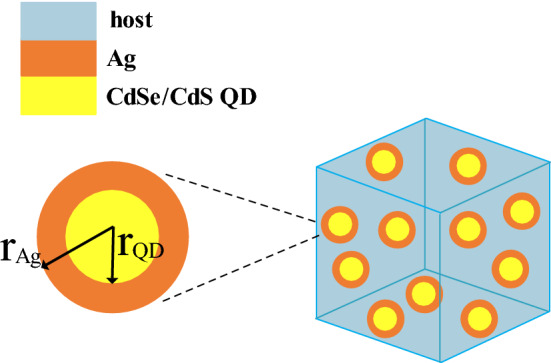
A sketch of the proposed low index layer obtained by dispersing silver-coated CdSe/CdS quantum dots within a polymer host. Here ${r}_{Ag}$ and ${r}_{QD}$ are the outer and inner radii of the nanoparticles respectively.

To analytically calculate the Effective permittivity, ${\varepsilon }_{eff}$ of the resultant composite medium, consisting of a polymer host medium and guest nano-particles with filling factor *f* (see Fig. 3), we use Clausius–Mossotti relationship^41^:

7 $$\frac{{\varepsilon }_{eff}-{\varepsilon }_{host}}{{\varepsilon }_{eff}+{2\varepsilon }_{host}}=f\frac{{\alpha }_{e}}{4\pi {{r}_{Ag}}^{3}}$$

where ${\varepsilon }_{host}$ is the permittivity of the host medium, *f* is the volume filling factor $f=4 N\pi {{r}_{Ag}}^{3}/3$ (where, *N* is the numberof particles per unit volume, and ${r}_{Ag}$ is the outer radius of nanoparticles ), and ${\alpha }_{e}$ is electric polarizability. If particles have small sizes compared to the incident wavelength, ${\alpha }_{e}$ will be directly proportional to the scattering coefficient ${a}_{1}$ and can be calculated as^42^:

8 $${\alpha }_{e}=\frac{6\pi i{a}_{1}}{{k}^{3}}$$

The scattering coefficient, ${a}_{1}$, of the spherical core/shell particle can be calculated using Mie theory^40,41^ as:

9 $${a}_{1}=\frac{{\psi }_{1}\left(y\right)\left[{\psi }_{1}^{{{\prime}}}\left({m}_{2}y\right)-{A}_{1}{\chi }_{1}^{\mathrm{^{\prime}}}\left({m}_{2}y\right)\right]-{m}_{2}{\psi }_{1}^{{{\prime}}}\left(y\right)[{\psi }_{1}\left({m}_{2}y\right)-{A}_{1}{\chi }_{1}({m}_{2}y)]}{{\xi }_{1}\left(y\right)\left[{\psi }_{1}^{{{\prime}}}\left({m}_{2}y\right)-{A}_{1}{\chi }_{1}^{{{\prime}}}\left({m}_{2}y\right)\right]-{m}_{2}{\xi }_{1}^{{{\prime}}}(y)[{\psi }_{1}\left({m}_{2}y\right)-{A}_{1}{\chi }_{1}({m}_{2}y)]}$$

where $y=k{r}_{Ag} (k$ and ${r}_{Ag}$ are wavenumber and outer radius of nanoparticle, respectively), ${m}_{2}$ is the ratio of refractive indices of the shell and host materials (defined as ${m}_{2}^{2}={\varepsilon}_{Ag}/{{\varepsilon}_{host}}$ ) , and ${\psi }_{1}, {\chi }_{1}, {\xi }_{1}$ are Ricatti-Bessel functions defined as ${\psi }_{1}\left(z\right)=z{j}_{1}\left(z\right),{\chi }_{1}\left(z\right)=-z{y}_{1}\left(z\right)$, ${\xi }_{1}\left(z\right)=z{h}_{1}^{\left(1\right)}(z)$, with ${j}_{1}\left(z\right), {y}_{1}\left(z\right), {h}_{1}^{\left(1\right)}(z)$ being the spherical Bessel functions of the first and the second kind, and Hankel function of the first kind, respectively. The coefficient ${A}_{1}$ is the scattering coefficient of a sphere nano-particle (containing only the core part) and is described as^7,35^:

10 $${A}_{1}=\frac{{m}_{2}{\psi }_{1}\left({m}_{2}x\right){\psi }_{1}^{\prime}\left({m}_{1}x\right)-{m}_{1}{\psi }_{1}^{{\prime}}\left({m}_{2}x\right){\psi }_{1}\left({m}_{1}x\right)}{{m}_{2}{\chi }_{1}\left({m}_{2}x\right){\psi }_{1}^{{\prime}}\left({m}_{1}x\right)-{m}_{1}{\chi }_{1}^{{\prime}}({m}_{2}x){\psi }_{1}\left({m}_{1}x\right)}$$

where $x=k{r}_{QD}$ ($with {r}_{QD}$ being the radius of the quantum dot), and ${m}_{1}$ is the ratio of refractive indices of the quantum dot and host materials (defined as ${m}_{1}^{2}={\varepsilon }_{QD}/{{\varepsilon}_{host}}$). For the permittivity of CdSe/CdS quantum dot, we have used the reported experimental results^39^, and for the relative dielectric constant of Silver, the developed corrected Drude model^38^ for thin films, $4.56-\frac{{{\omega }_{p}}^{2}}{({\omega }^{2}+i\gamma \omega )}$ with ${\omega }_{p}=1.4\times {10}^{16} {\mathrm{s}}^{-1}$ and $\gamma =1.0\times {10}^{14} {\mathrm{s}}^{-1}$, is used.

**Figure 4:**
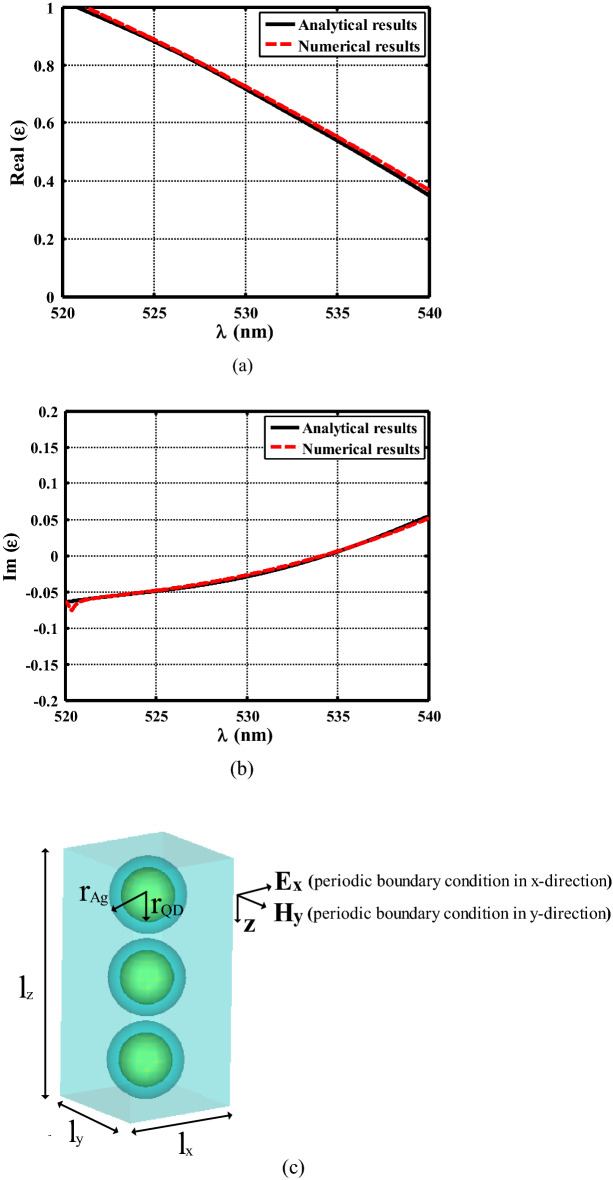
Real (**a**) and imaginary (**b**) parts of the effective permittivity of the composite medium including silver-coated CdSe/CdS quantum dots dispersed inside the polymeric host. (**c**) Simulation setup used to numerically retrieve the effective permittivity of the low index layer. ${r}_{QD}=4\,\,\mathrm{ nm}, {r}_{Ag}=5.8\,\,\mathrm{ nm}$, ${l}_{x}={l}_{y}=18\,\,\mathrm{ nm}$ and ${l}_{z}=38\,\,\mathrm{ nm}.$

Using (7)–(10), the effective permittivity of our designed medium with geometrical parameters of ${r}_{QD}=4\,\,\mathrm{ nm}, {r}_{Ag}=5.8\,\,\mathrm{ nm}$, $f=0.2$, is calculated and shown in Fig. 4 versus the operating wavelength. In Fig. 4, analytical results for the real and imaginary parts of the effective permittivity (solid lines) are compared with numerically extracted results (dash lines).

In order to numerically extract the effective parameters of the designed medium, a 3D full-wave numerical simulation is performed using CST software (details in the “Methods” section). The setup used in this simulation, is shown in Fig. 4c. As shown in this figure, a unit cell which contains a piece of the polymer host with silver-coated quantum dot particles inside, is shined by a plane wave propagating in the z direction, and the reflection and transmitted waves are used to retrieve the effective permittivity^43^. In this simulation, periodic boundary condition is used around the structure to mimic an infinite slab. The results of this simulation, are shown and compared with analytically calculated values in Fig. 4. As shown in this figure, there is a good agreement between theoretical predictions and numerical results ($\Delta \left|\varepsilon \right|\prec 0.01)$. According to the results of Fig. 4, at the wavelength of $\lambda =534\,\,\mathrm{ nm}$, we will have a material with $Real ({\varepsilon }_{eff})=0.55$, and ${Im (\varepsilon }_{eff})=0.003$, which can be considered as a material with the relative dielectric constant smaller than 1 and a neglectable loss.

## Numerical results and discussion

In this section, we use quantum dots proposed and analyzed in the previous section, to realize the proposed carpet cloak. The proposed carpet cloak consists of alternating low index and silicon layers, with the geometrical parameters and dimensions reported in Table 1. For the low index layer, here we use quantum dots distributed inside the polymer host as explained in the previous section.

Here, we perform full wave numerical simulations to verify the performance of the designed cloak (details in the “Methods” section). This simulation is carried out using Comsol Multiphysics, a commercial finite element-based solver. In this simulation, the structure is illuminated by a gaussian beam and the bottom boundary condition is defined as PEC (modeling the ground plane) and the three other boundaries are considered as scattering boundary conditions.

**Figure 5:**
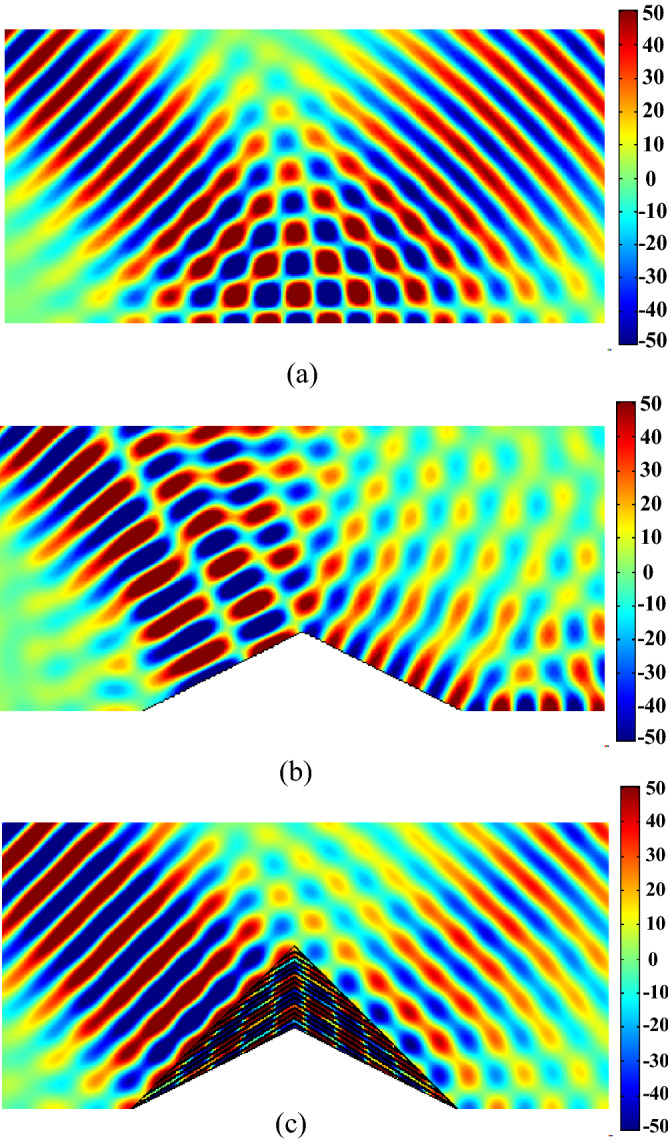
Numerically calculated magnetic field distributions of an incident light interacting with (**a**) a ground plane, (**b**) a bare conducting bump and (**c**) the bump covered by the proposed carpet cloak.

**Figure 6:**
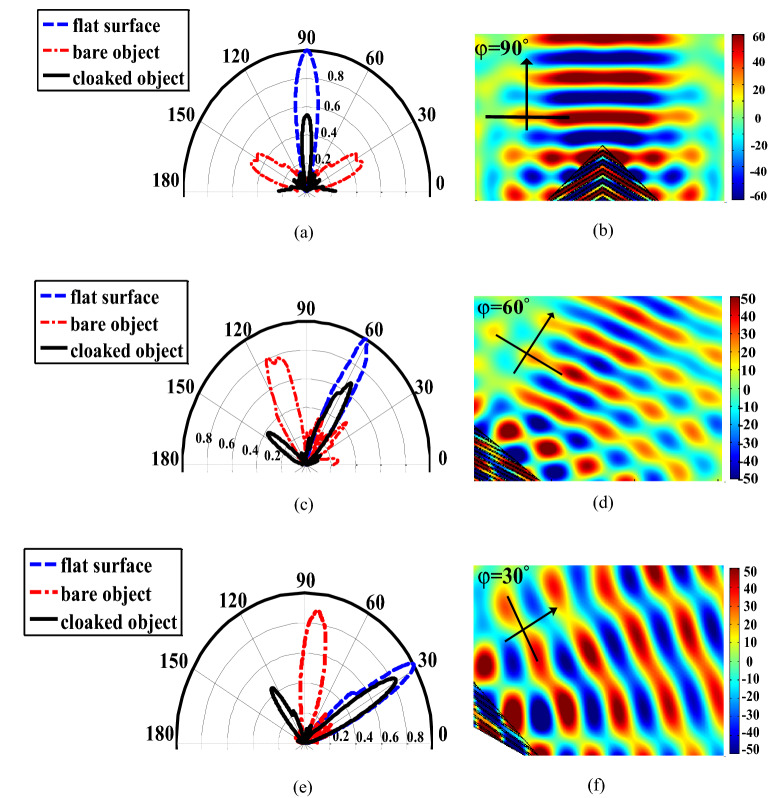
Far-field and near-field distribution of scattering fields for different incident angles of (**a**, **b**)$\varphi ={ 90}^{^\circ }$, (**c**, **d**) $\varphi ={120}^{^\circ }$, (**e**, **f**) $\varphi ={150}^{^\circ }$, at the free-space wavelength of 534 nm.

**Figure 7:**
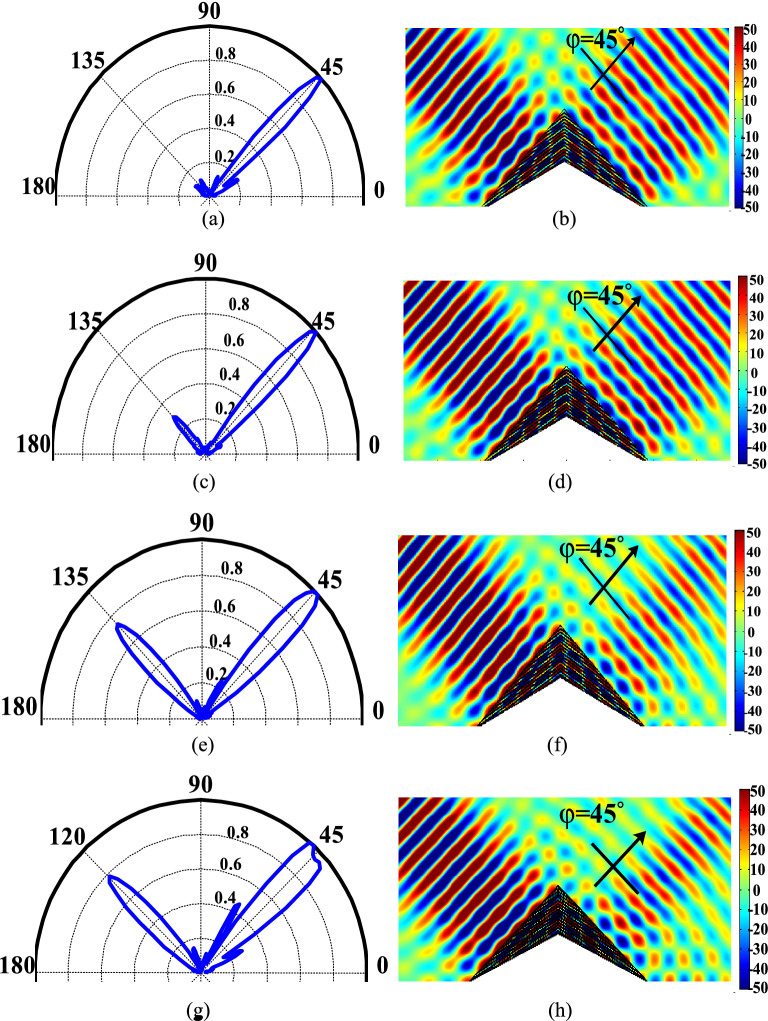
Numerically calculated far-field and near-field distribution of scattered fields for incident angle of $\varphi ={135}^{\circ }$ at the wavelength of (**a**, **b**) $\lambda =532\,\,\mathrm{ nm}$, (**c**, **d**) $\lambda =533\,\,\mathrm{ nm}$, (**e**, **f**)$\lambda =535\,\,\mathrm{ nm}$ and (**g**, **h**) $\lambda =536\,\,\mathrm{ nm}$.

The results of this simulation are shown in Figs. 5, 6 and 7. Figure 5 shows the simulation results at the operation wavelength of 534 nm and for the incident angle of ${45}^{^\circ }$. In this figure, the magnetic field distribution is shown for three different cases of a ground without any object on it (Fig. 5a), a bare triangular object on the ground (Fig. 5b), and the triangular object with the designed carpet cloak on it (Fig. 5c). As shown in Fig. 5a, when we have the flat ground, the light is reflected back at an angle exactly equal to the incident angle, as expected since the ground acts as a mirror. However, when the triangular object is placed on the ground, as shown in Fig. 5b, the scattered magnetic field becomes quite irregular due to the presence of the object which scatters light in different directions.

Figure 5c, on the other hand shows that when the designed carpet cloak is placed on the triangular object, the magnetic scattering field is confined highly in the specular direction, very similar to the results shown in Fig. 5a, for the flat ground without any object on it. Since the reflection is very similar to the flat ground, the observer assumes that there is no object on the ground and invisibility takes place.

However, as shown in Fig. 5, the waves scattered by the carpet cloak are somehow weaker than the waves scattered by the flat surface. This loss is due to the intrinsic loss of Silicon layers at the operation wavelength and can be reduced simply by using a material with lower loss such as SiO_2_. However, it should be noted that since SiO_2_ has a lower refractive index than Silicon at the operation wavelength, that selection will increase the height of the resultant cloak. Therefore, there is a trade-off here between the total loss of the cloak and its height.

Figure 6 shows the performance of the proposed carpet cloak for different incident angles of the illuminated light. In this figure, both near-field and far-field results are reported for incident angles of $\varphi ={90}^{^\circ },{120}^{^\circ },$ and ${150}^{^\circ }$. The near-field results, demonstrated in Fig. 6b, d, and f, show the scattered field from an object covered with the proposed carpet cloak. The far-field patterns of the scattering fields are illustrated in Fig. 6a, c, and e for different incident angles and three different cases of flat surface, bump (object without the cloak), and the object with the proposed carpet cloak on it. As shown in these figures, when there is no cloak (red curves), the response is very different from the response of a flat surface (blue curves). However, by using the proposed cloak (black curves), the response of the structure is much more similar to the response of a flat surface (blue curves) by having the main reflection in the same direction as that of the flat surface. For example, in Fig. 6a, for a bare object (red curves), instead of reflection in the normal direction which is desirable for normal incidence, we observe two main reflections in the directions of $\varphi ={30}^{^\circ }, {\varphi =150}^{^\circ }$. However, when using the proposed cloak (black curves) the reflection happens at $\varphi ={90}^{^\circ }$ as desired. The same phenomena are observed in Fig. 6d and f.

Results of Fig. 6 prove that the proposed structure operates well for incident angles in the range of ${30}^{\circ }<{\varphi <150}^{^\circ }$. However, for angles outside this range, the distribution of scattered field will be distorted, meaning that the scattered wave would not be similar to the one scattered from a flat surface. This can be explained through the increase in the impedance mismatch (which is a function of the incident angle) between the Air and the layered structure which makes most of the wave reflect back and only a small portion of it go through the layered structure and interact with it. The Results shown in Fig. 6c, and e, on the other hand show that at some incident angles, when using the proposed cloak, a backward scattering is observed. This backward scattering is one of the drawbacks of the carpet cloaks realized by layered structures which has been also reported in previous works^6,7^. The reason behind this backward scattering is the boundary impedance mismatching, on which we are working to reduce. However, it should be noted that this backward scattering is much weaker than the forward scattering as shown in Fig. 6c, e, and therefore, this does not mainly affect the performance of the developed cloak.

To investigate the frequency response of the proposed cloak, Fig. 7 illustrates the response of the cloak at four other wavelengths of 532 nm (frequency of 564 THz), 533 nm, 535 nm and 536 nm (frequency of 560 THz). Both near-field and far-field results are reported in this figure. As shown in this figure, the proposed cloak has a good performance at these wavelengths. However, our study shows that for the wavelengths outside this range, the proposed cloak does not work well. The reason behind this is that outside this range of wavelengths, the designed low index medium has an unneglectable loss. Therefore, to increase the operation bandwidth, quantum dots can be optimized to provide a low loss and low index at a wider range of frequencies.

It should be noted that in all the results shown in Figs. 5, 6 and 7, the simulations are carried out using the effective permittivity retrieved by CST (see Fig. 4), and the whole structure including real nanostructured composite is not simulated. The reason behind this, is that the simulation of the whole structure with precise nanoparticles is not possible using typical computational resources due to the numerous alternating layers and nanoparticles used in our design. To investigate the accuracy of our simulation results, we have carried out another numerical simulation (details in the “Methods” section). In this simulation, the Electromagnetic response of two structures is compared. One structure is a slab including numerous nanoparticles (36 ones in our case), and another one is a slab with the same size but including a material with effective permittivity shown in Fig. 4.

**Figure 8:**
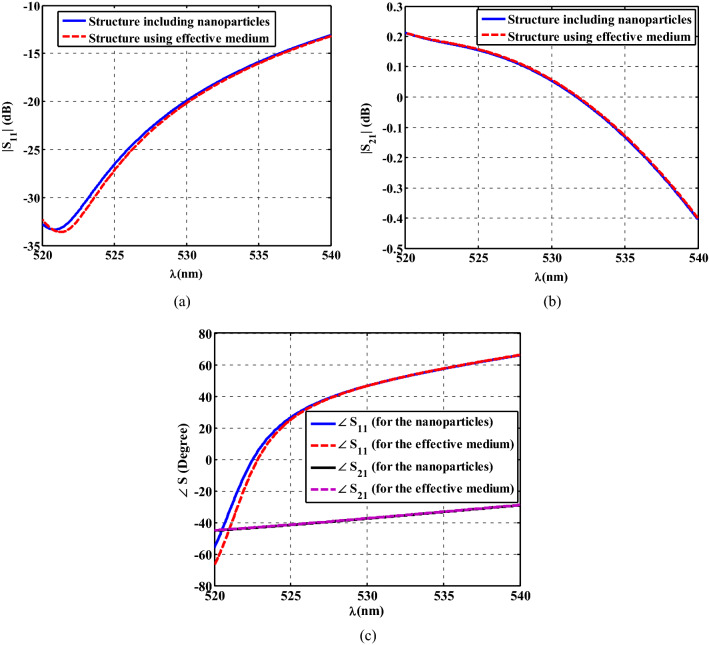
Simulation results of a slab containing real nanoparticles is compared with a slab with effective parameters. (**a**) Magnitude of S_11_, (**b**) magnitude of S_21_, (**c**) phase of S_11_ and S_21_.

Both slabs are shined by a plane wave propagating in the z direction, and their reflection and transmitted waves are compared with each other. The results of this comparison is shown in Fig. 8.

As shown in this figure, there is a great agreement between the S parameters (both magnitude and phase) achieved from these two structures. This comparison proves that using effective parameters is accurate enough for modeling layered structures and confirms the accuracy of the results shown in Figs. 5, 6 and 7.

## Conclusion

In this paper, a new method to reduce the profile of the multi-layered carpet cloaks was proposed, and investigated. It was analytically proved that using layers with the relative dielectric constants smaller than 1 in construction of carpet cloaks can remarkably reduce their thicknesses. Using the achieved analytical results, a low-profile carpet cloak using alternating layers of silicon and low index materials was designed with a height that was 0.4 times of the height of a traditional carpet cloak without using these layers. Low index layers were realized using silver-coated CdSe/CdS nanoparticles dispersed in a polymer host. In order to validate the analytical results, full-wave numerical simulations were performed. Numerical results showed that the proposed cloak operates well for a wide range of incident angles. It should be noted that the cloak designed in this paper was used only to prove the concept and is not an optimum structure. For example, by decreasing the permittivity of the low index layer further, shorter cloaks can be designed.

## Methods

In this paper, we have numerically and analytically investigated the performance of the proposed low-profile multi-layer carpet cloak using low index layers in order to reduce the thickness of the carpet cloak. The proposed carpet cloak simulation is carried out using Comsol Multiphysics. In this simulation, the carpet cloak is illuminated by a gaussian beam and the bottom boundary condition is defined as PEC (modeling the ground plane) and the three other boundaries are considered as scattering boundary conditions. In order to analyze the performance of the proposed low index slab consisting of silver-coated CdSe/CdS quantum dots dispersed in a polymer host with specific filling factor, a 3D full-wave numerical simulation is performed using CST software. The low index slab is shined by a plane wave propagating in the z direction and periodic boundary condition is used around the structure to mimic an infinite slab. The inner and outer radii of nanoparticles are ${r}_{QD}=4\,\,\mathrm{ nm}$ and ${r}_{Ag}=5.8\,\,\mathrm{ nm}$ respectively with filling factor $f=0.2$. In order to verify the accuracy of the simulations, we have compared electromagnetic responses of two structures including the slab using numerous nanoparticles (36 ones) and the slab using retrieved effective permittivity instead of real nano-particles. In order to retrieve the effective permittivity of the slab including numerous nanoparticles, we have used MATLAB software. Also, periodic boundary condition is used around the low index slab.

## Data Availability

The dataset used and/or analyzed during the current study are available from the corresponding author on reasonable request.
